# Recombinant Adeno-Associated Virus Serotype 6 (rAAV6) Potently and Preferentially Transduces Rat Astrocytes *In vitro* and *In vivo*

**DOI:** 10.3389/fncel.2016.00262

**Published:** 2016-11-11

**Authors:** Alexandra L. Schober, Dmitriy A. Gagarkin, Ying Chen, Guangping Gao, Lauren Jacobson, Alexander A. Mongin

**Affiliations:** ^1^Department of Neuroscience and Experimental Therapeutics, Albany Medical College, AlbanyNY, USA; ^2^ViGene Biosciences, Inc., RockvilleMD, USA; ^3^Horae Gene Therapy Center–Department of Microbiology and Physiological Systems, University of Massachusetts Medical School, WorcesterMA, USA

**Keywords:** adeno-associated virus, AAV2, AAV6, astrocytes, brain, *in vivo*

## Abstract

Recombinant adeno-associated virus vectors are an increasingly popular tool for gene delivery to the CNS because of their non-pathological nature, low immunogenicity, and ability to stably transduce dividing and non-dividing cells. One of the limitations of rAAVs is their preferential tropism for neuronal cells. Glial cells, specifically astrocytes, appear to be infected at low rates. To overcome this limitation, previous studies utilized rAAVs with astrocyte-specific promoters or assorted rAAV serotypes and pseudotypes with purported selectivity for astrocytes. Yet, the reported glial infection rates are not consistent from study to study. In the present work, we tested seven commercially available recombinant serotypes– rAAV1, 2, and 5 through 9, for their ability to transduce primary rat astrocytes [visualized via viral expression of green fluorescent protein (GFP)]. In cell cultures, rAAV6 consistently demonstrated the highest infection rates, while rAAV2 showed astrocytic transduction in some, but not all, of the tested viral batches. To verify that all rAAV constructs utilized by us were viable and effective, we confirmed high infectivity rates in retinal pigmented epithelial cells (ARPE-19), which are known to be transduced by numerous rAAV serotypes. Based on the *in vitro* results, we next tested the cell type tropism of rAAV6 and rAAV2 *in vivo*, which were both injected in the barrel cortex at approximately equal doses. Three weeks later, the brains were sectioned and immunostained for viral GFP and the neuronal marker NeuN or the astrocytic marker GFAP. We found that rAAV6 strongly and preferentially transduced astrocytes (>90% of cells in the virus-infected areas), but not neurons (∼10% infection rate). On the contrary, rAAV2 preferentially infected neurons (∼65%), but not astrocytes (∼20%). Overall, our results suggest that rAAV6 can be used as a tool for manipulating gene expression (either delivery or knockdown) in rat astrocytes *in vivo*.

## Introduction

Viral vectors are widely used for gene delivery and manipulation of gene expression within the CNS. Among them, adeno-associated viruses (AAVs) have become increasingly popular due to their non-pathogenicity, low propensity to induce innate immune responses, ability to effectively transduce dividing and non-dividing cells and produce long-lasting gene expression (Kaplitt et al., 1994; Wu et al., 2006b; Weinberg et al., 2013; Salganik et al., 2015). The efficacy of AAV constructs in basic and pre-translational studies led to their successful introduction into clinical practice, including applications in the CNS. Over the past decade, recombinant AAVs have been utilized for gene delivery in clinical trials targeting the brain, spinal cord, and retinal tissue (reviewed in Surace and Auricchio, 2008; Lentz et al., 2012; Weinberg et al., 2013; Bartus et al., 2014). The encouraging safety profile of rAAV human gene therapies, including those tested in the CNS, and the effective phenotypic correction in several human monogenic disorders reinforced the appeal of this approach and further increased the utilization of rAAVs in basic science studies.

Adeno-associated viruses are a small, non-enveloped viral species belonging to the *Dependovirus* genus within the family of parvoviruses. They require helper viruses, such as adenovirus or herpes virus, for successful replication. The simple AAV genome consists of two inverted terminal repeat regions (ITR) that flank the main open reading frames of the linear, single-stranded DNA totaling approximately 4.7 kb. The *rep* part of the genome encodes four proteins responsible for viral replication and site-specific chromosomal integration; an additional *cap* open reading frame is responsible for production of three different capsid proteins [reviewed in (Wu et al., 2006b; Daya and Berns, 2008; Salganik et al., 2015)]. In the recombinant AAVs used for research and clinical purposes, the *rep* and *cap* parts of the genome are removed and replaced with the ITR-flanked constructs encoding gene(s) of interest and/or shRNA-generating constructs, plus antibiotic-resistance genes that are required for selection. Therefore, packaging of recombinant AAVs requires additional plasmids encoding *rep* genes (typically derived from AAV2) and *cap* genes assorted from various AAV serotypes or genetically redesigned to modify capsid properties, as well as the helper plasmid providing adenoviral helper genes (Gao et al., 2005; Wu et al., 2006b; Kotterman and Schaffer, 2014).

The variability of AAV capsid proteins determines very strong differences between serotypes and pseudotypes in terms of their cell type and tissue tropism. The prototypical AAV2 gains access to target cells by binding to heparan sulfate proteoglycan and via additional interaction with co-receptors, such as integrins α_V_β_5_, α_V_β_1_, hepatocyte growth factor, and laminin (reviewed in Wu et al., 2006b; Daya and Berns, 2008; Asokan et al., 2012). Cellular surface receptors contributing to infection by other AAV serotypes are less studied, but thought to involve either sialic acid- or galactose-containing proteoglycans (Asokan et al., 2012). A very recent publication identified the orphan transmembrane protein KIAA0319L as a universal and indispensable AAV receptor for several common AAV serotypes, including AAV6, suggesting that further progress in our understanding of the mechanisms of cell entrance should be expected (Pillay et al., 2016).

Astrocytes are the most numerous and functionally complex cells in the CNS that critically contribute to normal brain functions and numerous neurological disorders (Sofroniew and Vinters, 2010; Parpura et al., 2012). With increased appreciation and evolving interest in normal and pathological roles for this class of glial cells, there is a growing demand for viral vectors effectively targeting astroglia (Merienne et al., 2013). Yet, the majority of past studies have focused on rAAV transduction of neuronal cells. The most frequently utilized serotype, rAAV2, targets neurons nearly exclusively in rodents and in non-human primates (see for example Kaplitt et al., 1994; Burger et al., 2004; Dodiya et al., 2010, and reviews by Tenenbaum et al., 2004; Weinberg et al., 2013). The same transduction patterns were reported for rAAV1, 5, 8, and 9 (Burger et al., 2004; Cearley and Wolfe, 2006; Dodiya et al., 2010; Markakis et al., 2010). In contrast, other publications identified moderate-to-strong tropism for astrocytes in studies with rAAV1, 5, 8, and 9 (Davidson et al., 2000; Foust et al., 2009; Gray et al., 2011; Aschauer et al., 2013; Petrosyan et al., 2014; Watakabe et al., 2015), or even preferential astrocytic transduction by rAAV8 and rAAV-rh43 (Lawlor et al., 2009; Aschauer et al., 2013). The reasons for variability in glial tropism of AAV serotypes are still poorly understood (see “Discussion” for further details). Some refinements in glial specificity have been made with incorporation of the astrocyte-specific promoters, such as gfa2 or gfaABC1D (Merienne et al., 2013; Weinberg et al., 2013). However, even with these improvements, astrocytic transduction rates appear to be moderate and new AAV vectors targeting astroglial cells would be a welcome addition to the field. Here, we report that one of the less studied rAAV serotypes, rAAV6, potently and preferentially targets astrocytes in the rat cortex.

## Materials and Methods

### Materials and Reagents

Bovine serum albumin (BSA), 4′,6-diamidino-2-phenylindole dihydrochloride (DAPI), *N*-propyl-gallate, Triton X-100, all salts and solvents were purchased from Sigma–Aldrich (St. Louis, MO, USA), unless otherwise specified, and were of the highest purity available. Cell culture media, sera, antibiotics of the Gibco^®^ brand, and the recombinant protease TrypLE^®^ were acquired from Life Technologies/Thermo Fisher Scientific (Waltham, MA, USA). The sources and catalog numbers for all used antibodies are provided as they appear first in the text.

### rAAV Vectors

Recombinant AAVs – serotypes rAAV1, rAAV2, rAAV5, rAAV6, rAAV7, rAAV8, and rAAV9 – were acquired from ViGene Biosciences (Rockville, MD, USA). Each of these viral constructs were prepared using triple-plasmid transfection of HEK293 cells. The main pAV-U6-GFP plasmid contained AAV2 ITRs, the shRNA insertion site under control of the U6 promoter, a sequence encoding enhanced green fluorescent protein (EGFP) under control of the distinct CMV promoter, and the ampicillin resistance gene. Separate Rep/Cap plasmid and the helper plasmid provided components of the viral replication machinery and the capsid proteins of selected AAV serotypes. After HEK293 cell lysis, rAAV particles were purified by iodixanol gradient ultracentrifugation. Further details about these viruses and their packaging and purification can be found on the company website^1^. As an additional control, we also used rAAV1, rAAV2, rAAV5, and rAAV9 that were packaged at the University of Massachusetts Medical School (Worcester, MA, USA) according to the previously published method (Gao and Sena-Esteves, 2012). The latter rAAVs were also generated by a triple-plasmid transfection of HEK293 cells. These constructs encoded EGFP under the control of the promoter containing the cytomegalovirus immediate-early enhancer (CMV IE) and 260 nucleotides (-1261 to -1,001) of the chicken β-actin 6 promoter, and their efficacy was validated *in vivo* in a recent study (Vincent et al., 2014). The rAAVs were packaged with assorted capsid proteins of the specified serotypes. Viral particles were purified by CsCl gradient ultracentrifugation. At both facilities, rAAVs were titered by quantitative polymerase chain reaction (qPCR) and their purity was further verified by Coomassie Brilliant Blue or silver protein staining after separation on SDS-polyacrylamide gels.

### Cell Cultures of Primary Rat Astrocytes and Retinal Pigmented Epithelial Cells (RPE)

Primary astrocyte cultures were prepared from the brains of 1–2-day old Sprague–Dawley rat pups as previously described (Mongin et al., 2011). All animal procedures strictly conformed to NIH Guidelines for Care and Use of Laboratory Animals and were approved by the Institutional Animal Care and Use Committee of the Albany Medical College. In brief, rat pups were killed by rapid decapitation and their brains harvested. The cortices were separated from the meninges and hippocampi, and placed into ice-cold Opti-MEM. The brain tissue was minced and enzymatically dissociated in a solution containing equal parts of the recombinant protease TrypLE and Opti-MEM, and additionally supplemented with DNase I (1 mg/ml). Cell extraction with TrypLE was repeated three times at 37°C. The first extraction was discarded. Dissociated cells from the last two extractions were combined and sedimented by brief centrifugation at 1,000 × *g*. Cells were re-suspended in Dulbecco’s modified Eagle minimum essential medium (DMEM) containing 10% of heat inactivated horse serum (HIHS), 50 U/mL penicillin, and 50 μg/mL streptomycin, and then plated on poly-D-lysine coated T-75 culture flasks at the density of 200,000 cells per flask. The primary astrocyte cultures were grown in DMEM-10% HIHS plus antibiotics for 2–4 weeks in a humidified atmosphere of 5% CO_2_/balance air at 37°C. Cell culture media were changed twice per week. Culture purity was >95% as periodically verified by staining with antibody recognizing the astrocytic marker glial fibrillary acidic protein (anti-GFAP, Sigma–Aldrich, catalog #G3893).

Human RPE (ARPE-19, gift of Dr. Sally Temple, New York Neural Stem Cell Institute, Rensselaer, NY, USA, passage unknown) were used as an additional control for AAV efficacy. They are known to be readily transduced with a variety of AAV serotypes and pseudotypes (Surace and Auricchio, 2008). ARPE-19 were cultivated in DMEM supplemented with 10% of fetal bovine serum (FBS) and antibiotics in T75 flasks as described above. They were periodically passaged after detachment with TrypLE.

### *In vitro* rAAV Infection and Immunocytochemical Procedures

Primary astrocytes or ARPE-19 cells were plated in poly-D lysine coated 24-well plates with glass bottoms at a sub-confluent density and transduced with seven different rAAV vectors. Based on the pilot titration experiments, we selected a viral titer of 10^5^ genome copies per cell (gc/cell) to produce near maximal infection rates. Cells were incubated with individual rAAVs in Opti-MEM at 37°C. After the initial 4-h incubation, each well was supplemented with an equal volume of DMEM-10% HIHS for astrocytes or DMEM-10% FBS for ARPE-19. After an additional 20 h, rAAV-containing media were replaced with fresh DMEM-HIHS or DMEM-FBS as appropriate. Viral transduction rates (GFP expression) were determined after 48–72 h.

To quantify transduction rates, cells were washed twice from culture media with phosphate-buffered saline (PBS) and fixed at room temperature (20°C) using 4% paraformaldehyde in PBS. After fixation they were washed again twice with PBS and permeabilized with 0.1% Triton X-100 in PBS for 15 min at room temperature. After two additional washes with PBS, cells were blocked with 1% BSA in PBS for 1 h at room temperature. For immunostaining, fixed astrocytes were incubated 1 h at room temperature with either rabbit polyclonal anti-GFP antibody (Life Technologies/Thermo Fisher Scientific, catalog # A6455, 1:2,000) or chicken polyclonal anti-GFP antibody (Life Technologies/Thermo Fisher Scientific, catalog #A10262, 1:500 dilution). Unbound primary antibodies were removed by three 5-min washes in PBS, and cells were incubated with goat anti-rabbit secondary antibody conjugated to Alexa–Fluor 488 (Life Technologies/Thermo Fisher Scientific, A11008, 1:1,000 dilution) or goat anti-chicken secondary antibody conjugated to Alexa–Fluor 488 (Life Technologies/Thermo Fisher Scientific, cat. # A11039, 1:500 dilution) for 1 h at room temperature. The excess of secondary antibodies was removed by three 5-min washes with PBS, and cells were then counterstained with DAPI (10 μg/ml) for 15 min at room temperature and washed once with PBS. Alexa-488 fluorescence images were captured with a Zeiss LSM510META confocal microscope (Carl Zeiss, Oberkochen, Germany). To ensure validity of the comparisons of GFP expression, all images were taken at identical exposure and settings.

### Animal Surgeries and rAAV Injection

Stereotaxic injection of rAAV constructs was performed under anesthesia according to the procedure approved by the Institutional Animal Care and Use Committee of the Albany Medical College in strict adherence to NIH Guidelines for Care and Use of Laboratory Animals. Five-week-old male Sprague–Dawley rats weighing approximately 200 g were anesthetized with isoflurane (5% for induction, 2% for maintenance) in 30% O_2_/balance N_2_. Body temperature was monitored via a rectal probe and maintained at 36.5–37°C with a heating pad. Animals were placed in a rodent stereotaxic frame (David Kopf Instruments, Tujunga, CA, USA). Their scalp was additionally anesthetized with subcutaneous local injection of Bupivacaine (Hospira, Lake Forest, IL, USA) and a small scalp incision was made to locate the bregma. Next, a small burr hole in the skull just above the barrel cortex region (-2 mm anterior, +5 mm lateral from the bregma) was made using a Microtorque II drill (Harvard Apparatus, Holliston, MA, USA). A Hamilton syringe containing either rAAV2 or rAAV6 was lowered 2.1 mm below the dura and 1 μL of virus was injected at the rate of 0.2 μL/min. After a 10-min wait period to ensure proper diffusion of virus, the syringe was drawn 1 mm up and the second 1-μL injection was made. The incision was closed using staples, 0.1 mg/kg Buprenorphine (Reckitt Benckiser Healthcare, Hull, UK) was injected subcutaneously, and triple antibiotic ointment was applied to the closed wound. The animals were additionally given Buprenorphine twice daily for two days following the surgery. They were kept for an additional 3 weeks to allow for spread of the virus and expression of the marker protein GFP before harvesting the brain.

### Immunohistochemical Evaluation of Viral Transduction

To assess viral transduction, we performed immunohisto chemical analysis of the rAAV-driven GFP expression. Rats were given a lethal injection of sodium pentobarbital and perfused intracardially first with a heparin solution in PBS, followed by 4% paraformaldehyde solution in PBS. Brains were removed and post-fixed in 4% paraformaldehyde for 24 h at 4°C, then transferred into a 30% sucrose solution (4°C) for cryoprotection. After completing cryoprotection, brains were mounted on a cryostat stage using Tissue-Tek O.C.T. compound (Sakura Finetek, Torrance, CA, USA), frozen to -20°C, and sectioned into 25-μm sections using a Leica CM3050 cryostat (Leica, Buffalo Grove, IL, USA). Sections were stored at -20°C in a cryoprotectant solution containing 50% Tris buffered saline (TBS), 30% ethylene glycol, and 20% glycerol, until the staining procedure.

Prior to staining, sections were washed five times for 5 min with TBS and blocked for 30 min at room temperature in a solution containing 3% normal goat serum (Vector Laboratories, Burlingame, CA, USA) and 0.3% Triton X-100. They were next incubated for 24–48 h at 4°C with rabbit polyclonal anti-GFP antibody (Life Technologies/Thermo Fisher Scientific, catalog # A6455, 1:400) diluted in 3% normal goat serum. Next, the slices were washed five times for 5 min with TBS and then incubated for 2 h at room temperature with goat anti-rabbit Alexa–Fluor 488 secondary antibody (Life Technologies/Thermo Fisher Scientific, A11008, 1:400) diluted in 3% normal goat serum. To identify which cell types were infected with AAV, we subsequently stained the same sections with either the mouse monoclonal anti-NeuN antibody (EMD-Millipore, cat. # MAB377, 1:500) or the mouse monoclonal anti-GFAP antibody (Sigma Aldrich, cat. # G3893, 1:400). The staining procedure was identical to that described for GFP, except that GFAP or NeuN immunoreactivity was visualized with goat anti-mouse secondary antibody conjugated to Alexa–Fluor 555 (Life Technologies/Thermo Fisher Scientific, cat. # A21422, 1:400). The excess of secondary antibody was removed with three additional 5-min washes with TBS. Brain sections were then counterstained with 0.1 μg/mL of DAPI at room temperature for 15 min, washed once with TBS, then mounted on microscope slides with the anti-fade reagent *N*-propyl-gallate and sealed with clear nail polish.

To ensure specificity of the signal, we periodically reversed the order of application for primary antibodies and noticed that when GFP antibodies were applied last, the resulting GFP signal was stronger. Also, adding 0.3% Triton X-100 into solutions of primary antibodies allowed for better antibody penetration into the sections. It should be stressed that the quantitative analyses of cellular tropism were performed under all of the above described scenarios, and changes in the order of antibody application did not impact conclusions on transduction efficacy or cellular tropism. GFP (Alexa-488), GFAP and NeuN (Alexa-555), and DAPI immunofluorescence images in the brain sections were captured using a Zeiss LSM-510 Meta confocal microscope at 250× magnification with the identical exposure and settings. Half-brain images were taken using a Leica TCS SPE confocal microscope at 15× magnification to visualize the full extent of viral spread. A series of six individual images were stitched together to make one representative image using the ImageJ stitching software.

### Data Analysis

For quantitative analysis of *in vivo* viral transduction, representative immunohistochemistry images were “coded” and analyzed by two investigators, who were blinded to the type of treatment (DG and AM), and additionally by the primary investigator (ALS). The individual cell counts for each stained section/IHC image were averaged among the three investigators and then the average values of cell counts for each immunohistochemical marker and the % fractions of the virus-infected neuronal and astroglial cells were statistically compared between treatment groups. Number of analyzed sections, injected animals, and total cell counts are given in the text and figure legends. The statistical comparisons between groups were performed using ANOVA and Tukey *post hoc* test for multiple comparisons in Origin 8.1 software suite (OriginLabs, Northampton, MA, USA).

## Results

### Among Seven Commonly Used rAAV Serotypes, rAAV6 Has High Tropism for Rat Primary Astrocytes *In vitro*

**FIGURE 1:**
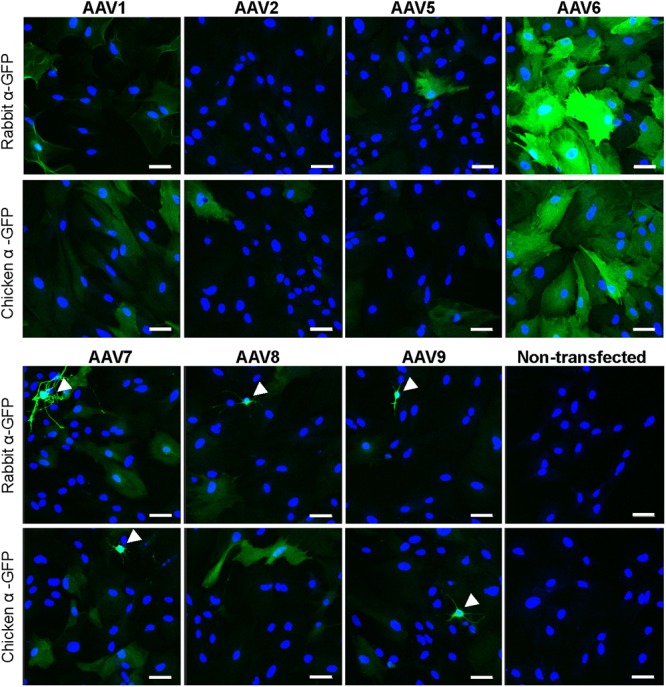
**Transduction of primary rat astrocytes with seven different rAAV-green fluorescent protein (GFP) viruses.** Astrocytes were transduced with 10^5^ gc/cell of either rAAV1, rAAV2, rAAV5, rAAV6, rAAV7, rAAV8, or rAAV9, or treated identically without the addition of virus (non-transfected), for 48 h. Results for rAAV1, rAAV2, rAAV5, and rAAV6 are shown in the top two rows and results for rAAV7, rAAV8, rAAV9 and non-transfected astrocytes are shown in the bottom two rows. Cells were stained with either rabbit or chicken anti-GFP antibodies, as indicated, and counterstained with 4′,6-diamidino-2-phenylindole dihydrochloride (DAPI) to visualize GFP-negative cells. Images were acquired using a confocal Zeiss LSM510 META microscope at 250× magnification, in the same cell preparation with identical exposure settings. Scale bar = 50 μm. Arrowheads point to cells with non-astrocytic morphology that were transduced with rAAV. Results are representative of at least nine wells and four independent experiments per transduction condition.

In order to identify an AAV serotype that effectively infects astrocytes, we tested several commercially available recombinant AAV vectors: rAAV1, rAAV2, rAAV5, rAAV6, rAAV7, rAAV8, and rAAV9, all of which expressed the GFP protein under the CMV promoter. In the first series of experiments, only rAAV6 demonstrated consistent and high levels of astrocytic transduction as detected with two different anti-GFP antibodies (**Figure 1**). As a specificity control, we immunostained non-transfected cells with the same two antibodies and detected no immunoreactivity (**Figure 1**). Other tested rAAV serotypes showed much lower levels of transduction with few GFP-positive astrocytes and much lower fluorescence levels in individual cells (**Figure 1**). Interestingly, rAAV7 and rAAV9, had an apparent tropism toward secondary cell types (such as microglia and O2A progenitors), which are present in primary astrocyte cultures in small numbers (arrowhead labels in **Figure 1**). To ensure that these results are replicable across different viral preparations, we first performed the same experiment with a second batch of viruses from the same vendor. In these latter experiments, we observed high GFP expression in rAAV6- but also in rAAV2-transduced astrocytes, pointing to differences between viral batches (**Supplementary Figure S1**). Next, we used an alternative set of viruses from a different packaging facility and with a different promoter, and found high-to-moderate levels of transduction with rAAV2 (AAV2/2) serotype, and rAAV1 (AAV2/1) and rAAV5 (AAV2/5) pseudotypes, respectively (**Supplementary Figure S1**). Due to a limited amount of viral constructs, the results with AAV2/2, AAV2/1, and AAV2/5 have not been repeated in several cell cultures, and no alternative batches of these viruses were tested.

**FIGURE 2:**
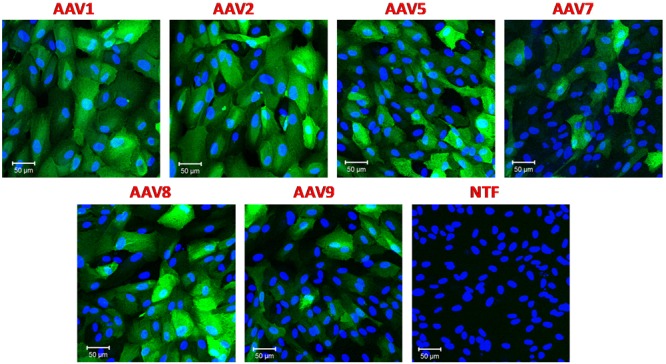
**Transduction of immortalized human retinal pigmented epithelial cell line (ARPE-19) with six different rAAV serotypes.** ARPE-19 cells were transduced with 10^5^ gc/cell of either rAAV1, rAAV2, rAAV5, rAAV7, rAAV8, or rAAV9, as indicated, for 72 h. Infected cells were visualized with rabbit anti-GFP antibodies, and counterstained with DAPI. Control non-transfected cells (NTF) were processed at the same time and in the same fashion. Images are representative of three replicates and were captured using Zeiss LSM510 META confocal microscope at identical exposure settings at 250× magnification. Scale bars = 50 μm.

All experiments mentioned above were performed using the same virus per cell ratio (10^5^ genome copies/cell). Since we saw dramatically different levels of transduction, we tested the rAAV1, rAAV5, rAAV7-9 serotypes in the RPE line ARPE-19 to ensure that they were fully functional. Retinal epithelial cells are known to be readily transduced with a variety of AAV serotypes and pseudotypes (Surace and Auricchio, 2008). We utilized the same batches of rAAV and the same virus/cell ratio as in the experiments presented in **Figure 1**. In strong contrast to astrocytes, ARPE-19 cells demonstrated very high levels of GFP expression with rAAV1, 2, 5, 8, and moderate infection rates with rAAV7 and rAAV9 (**Figure 2**).

Altogether, *in vitro* experiments indicate that all viral constructs, which were utilized in the present work, were fully functional and that only rAAV6 consistently demonstrated strong tropism toward astrocytes *in vitro*. Some but not all batches of rAAV2, rAAV1, and rAAV5 also showed moderate levels of astrocytic transduction. For rAAV2, differences were observed even for different batches obtained from the same packaging facility.

### Effective *In vivo* Delivery of rAAV6 and rAAV2 in the Rodent Cortex

**FIGURE 3:**
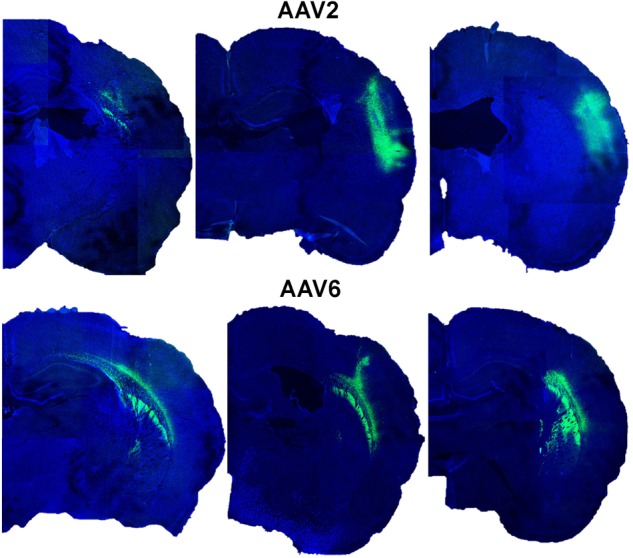
**Transduction of GFP expression by rAAV2 and rAAV6 after injection in rat cortex.** Representative images from six different animals were captured in proximity to the rAAV injection sites. Rats were injected with either rAAV2 (top row) or rAAV6 (bottom row) as described in “Materials and Methods.” Three weeks after injection, the brains were processed and 25-μm coronal sections were stained with anti-GFP antibody and counterstained with DAPI. Fluorescence signals were captured using a Leica TCS SPE confocal microscope at 15× magnification and a series of six individual fields per each brain were stitched together using the ImageJ software.

We next tested if efficient astrocytic transduction by rAAV6 also occurred *in vivo*. We used rAVV2 as an additional control because rAAV2 has been previously reported to preferentially transduce neurons *in vivo* (reviewed in Tenenbaum et al., 2004; Weinberg et al., 2013). As expected, our results showed that rAAV2 produced low levels of astrocytic infection in cell cultures. We injected rAAV6 or rAAV2 into the barrel cortex of male Sprague–Dawley rats as described in the “Materials and Methods.” It should be stressed that the same batches of virus were used for all *in vitro* and *in vivo* experiments to avoid potential impacts from inter-batch variability. The extent and localization of viral transduction in the brain was visualized in 25-μm coronal sections using immunohistochemistry with an anti-GFP antibody and documented using confocal microscopy. rAAV6 injection produced significant cell transduction, and the virus tended to spread away from the site of injection (see **Figure 3** for serotype differences and the variability of the viral spread within each serotype). The rAAV6-transduced GFP signal was detected up to 2 mm anterior and 2 mm posterior from the site of injection (**Supplementary Figure S2**). rAAV2 also induced robust cell transduction, but the viral signal tended to concentrate closer to the site of injection (**Figure 3**), and did not propagate further than 1–2 mm in the anterior-posterior direction (**Supplementary Figure S2**). Although quantification of the total volume of the rAAV-infected tissue was not one of the objectives of this work, these latter results are consistent with the literature reports indicating that rAAV2 spread in the rodent and the primate brains is much smaller than those for other rAAV serotypes (Burger et al., 2004; Taymans et al., 2007; Aschauer et al., 2013; Watakabe et al., 2015).

Another significant difference between rAAV6 and rAAV2 was in the morphology of the infected cells. rAAV6 targeted nearly exclusively astrocyte-like cells with smaller bodies and highly branched, short processes (right panels in **Supplementary Figure S3**). In contrast, rAAV2 targeted predominantly neuron-like cells with larger cell bodies and long polarized projections (left panels in **Supplementary Figure S3**), consistent with its known affinity toward neurons (Tenenbaum et al., 2004; Weinberg et al., 2013). In sum, both rAAV6 and rAAV2 successfully infected cortical brain tissue, and the rAAV-driven GFP expression persisted for at least 3 weeks.

### In the Rat Brain, rAAV6 Preferentially Transduces Astrocytes While rAAV2 Targets Neurons

**FIGURE 4:**
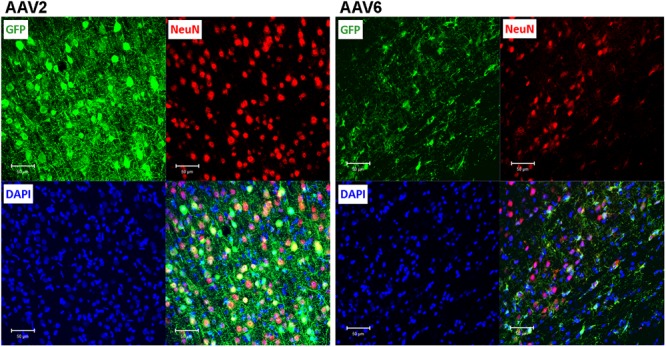
**Assessment of the ability of rAAV2 and rAAV6 to transduce neurons in the rat cortex.** Slices were stained for GFP as a marker of viral transduction (green), the neuronal marker NeuN (red), and DAPI (blue). The bottom right images in each set show overlay of all three signals, with shades of yellow representing colocalization (at this magnification it is not obvious in some cells due to differences in signal intensities and cellular location). Z-stack images were taken using Zeiss LSM510 META confocal microscope at 250× magnification and identical exposure settings. Scale bars = 50 μm. Images are representative of at least 12 sections from four different rats for each virus.

**FIGURE 5:**
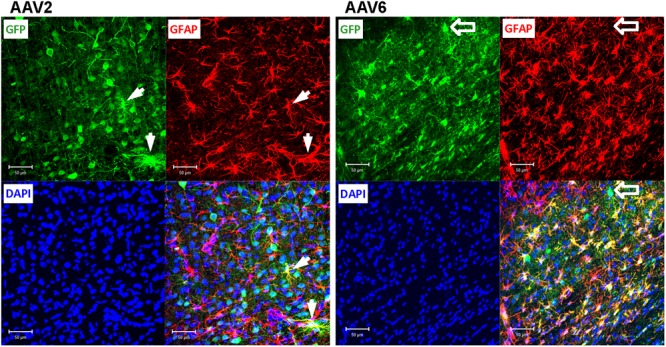
**Astrocytic tropism of rAAV2 and rAAV6 in the rat cortex.** Slices were stained for GFP as a marker of viral transduction (green), the astrocytic marker GFAP (red), and DAPI (blue). White arrows point to a few rAAV2-infected astrocytes (GFP+/GFAP+ cells for rAAV2). Although the vast majority of the AAV6 transduced cells were GFAP-positive, block arrows point to an exception, the rAAV6-transduced neuron-like cell (GFP+/GFAP- cell). Images were taken using a Zeiss LSM510 META confocal microscope at 250× magnification. Scale bars = 50 μm. Images are representative of at least 12 sections from four different rats for each virus.

To elucidate which specific cell types were infected by rAAV6 and rAAV2, we double-stained additional brain sections for the virally transduced GFP expression and either the neuron-specific marker NeuN or the astrocyte-specific marker GFAP. As expected, rAAV2 clearly transduced neuronal cells based on their NeuN immunoreactivity and morphology (GFP+/NeuN+ cells in **Figure 4**, AAV2 fields). In striking contrast, rAAV6 produced little to no signal in NeuN+ cells (**Figure 4**, AAV6 fields). The opposite was true, when virus-infected brains were stained for the astrocytic marker GFAP. In rAAV2-infected animals, we found very few GFP+/GFAP+ cells (**Figure 5**, AAV2 fields). In the rAAV6-transduced brains, there was a nearly perfect overlap between GFP and GFAP signals (**Figure 5**, AAV6 fields). These data strongly suggest that rAAV2 has a clear preference for neurons, while rAAV6 demonstrates strong tropism toward astrocytes.

**FIGURE 6:**
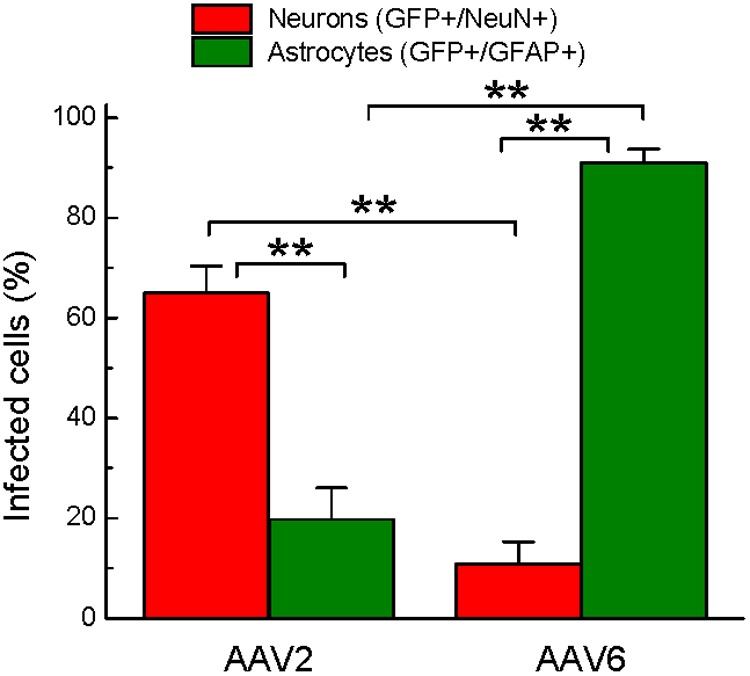
**Quantification of cell types transduced by either rAAV2 or rAAV6.** The data represent blinded analysis of randomly selected stained sections from rat brains transduced with either rAAV2 or rAAV6. The data are pooled from experiments using two different virus batches and four different animals for each rAAV serotype. Colocalization of GFP and NeuN signals was considered neuronal. Colocalization of GFP and GFAP signals was defined as astrocytic. The data were analyzed by ANOVA with *post hoc* Tukey test for multiple corrections and are presented as the means ± SEM (a total of 25 sections, 3+ different sections per brain, from four animals per viral construct). ^∗∗^*p* = < 0.01 between neurons and astrocytes transduced by the same serotype or between the same cell type transduced by different serotypes.

To quantify the cell tropism information, we performed blinded counts of GFP+/NeuN+ and GFP+/GFAP+ cells in 5–8 representative microscopic fields at 250× magnification (25× objective) in four animals injected with rAAV2 vs. four animals injected with rAAV6. Two different viral batches of each tested serotype were acquired from the same packaging facility to ensure reproducibility of the data. In the rAAV2-transduced brains, our counts identified 64.9 ± 5.5% of virus-infected (GFP+) cells as neurons (218/336 cells), based on the NeuN colocalization with GFP, while only 19.8 ± 6.3% of the infected cells were GFP+/GFAP+ astrocytes (58/284 cells, **Figure 6**). The majority of the remaining GFP+/NeuN- cells were likely neurons too, based on their morphology (neuron-like larger cell bodies and long processes, similar to those seen in **Figure 4**, AAV2 fields). One possible reason for the apparent underestimation of the rAAV2-transduced neurons is weak NeuN immunoreactivity in a number of stained sections, which made it difficult for blinded investigators to reliably identify double-positive cells.

Conversely, the rAAV6 viral construct transduced primarily GFAP+ astrocytes. Cell counts established that 90.9 ± 2.8% of GFP+ cells were astrocytes (241/265 counted cells, **Figure 6**, and representative image in **Figure 5**, AAV6 fields). When the AAV6-infected brains were counterstained with NeuN, we found that only 10.8 ± 4.4% of GFP-expressing cells were neurons (14/128 counted cells, **Figure 6** and representative image in **Figure 4**, AAV6 fields). The high tropism of rAAV6 for astrocytes was consistently seen in four different animals injected with two different batches of the virus. Counts of neurons and astrocytes transduced by rAAV2 and rAAV6 differed significantly (**Figure 6**).

## Discussion

The major finding of this study is that the commercially available rAAV6 has high tropism for rat astrocytes *in vitro* and *in vivo* and can fairly selectively transduce astrocytes in the rat cortex. Among the seven tested rAAV serotypes only rAAV6 strongly and consistently transduced astrocytes in primary cell cultures. *In vivo*, rAAV6 transduced >90% of GFAP+ astrocytes in the infected brain areas, with minimal infection of neuronal cells. This is significant because the majority of currently available AAV constructs do not readily target astroglial cells.

As already mentioned in the “Introduction,” the extensive existing literature points to preferential neuronal tropism for majority of rAAVs. Up to 95% of rAAV2-transduced cells are typically counterstained with neuronal markers, and very few are identified as astrocytes or other classes of glia (see “Introduction” for references). Our present rAAV2 results are consistent with the prior findings. Similar preferential neuronal transduction has been reported in the CNS of rodents and non-human primates upon infusion of recombinant AAV1, AAV5, AAV8, and AAV9 (see for example Wang et al., 2003; Cearley and Wolfe, 2006; Taymans et al., 2007; Lawlor et al., 2009; Dodiya et al., 2010). Yet, for unknown reasons, the findings of the preferential neuronal tropism for rAAVs are not always consistent even within the same serotype. Moderate-to-significant transduction of glial cells, predominantly astrocytes, was seen in rodents and non-human primates injected with rAAV1, rAAV5, rAAV8, rAAV9, but also with rAAV-rh10, and rAAV-hu11 (Davidson et al., 2000; Foust et al., 2009; Aschauer et al., 2013; Petrosyan et al., 2014; Watakabe et al., 2015). AAV-rh43 was found to produce fairly selective astroglial infection (Lawlor et al., 2009), and further improvements in astrocytic selectivity have been accomplished using rAAV5, rAAV8, and rAAV-rh43, which expressed gene constructs under the control of the astrocytic promoter Gfa2 (Lawlor et al., 2009; Drinkut et al., 2012; Merienne et al., 2013). Discrepancies between glial tropism of rAAV constructs in different studies may be due to a number of reasons, including reported variability amongst brain regions, with animal age, and species (Burger et al., 2004; Foust et al., 2009; Aschauer et al., 2013; Weinberg et al., 2013). Furthermore, even the methods of packaging and purification may have a strong impact on the viral infectivity and astrocytic tropism (Klein et al., 2008). Potential effects on cell type tropism from different universal promoters and other integrated genetic elements are also possible but have not been systematically studied.

Here, we report high astrocytic transduction rates and fairly strong astrocyte selectivity for a relatively understudied AAV serotype, rAAV6. Few previous publications, which compared different AAVs, have shown that rAAV6 has the propensity to successfully transduce brain tissue in mice, rats, and primates (Koerber et al., 2009; Blits et al., 2010; Markakis et al., 2010; Aschauer et al., 2013). rAAV6 affinity for astrocytes appears to vary between brain regions from low to relatively high (Koerber et al., 2009; Aschauer et al., 2013). In a separate study, rAAV6 constructs have also been used to infect malignant cells of human glioblastoma multiforme, which arise from astrocytes (Huszthy et al., 2005). In contrast to the brain, rAAV6 has been better characterized in peripheral tissues, and demonstrated a high capacity for transducing airway epithelial cells, cardiac tissue, and skeletal muscles (Halbert et al., 2001; Blankinship et al., 2004; Palomeque et al., 2007). Overall, the existing literature contains precedents for astrocytic transduction by rAAV6, but has not provided conclusive evidence of the relative tropism of this serotype for neurons and astrocytes.

What factors may determine rAAV6 cell type tropism? The bulk of our experiments have been carried out with rAAVs, which differed only in their capsid proteins and were packaged and purified in an identical manner at the same facility. Therefore, the differences in astrocytic tropism are likely determined by capsid protein structure. Although we did not pursue further the molecular underpinnings for this phenomenon, some inferences may be made based on the already existing literature. Several studies found that AAV1 and AAV6, which have structurally similar (∼99% homologous) capsid proteins, use α2,3 and α2,6 *N*-linked sialic acid moieties of the cell surface proteoglycans for entry (Wu et al., 2006c; Huang et al., 2016). In the present work, we found a strong dissimilarity between rAAV1 and rAAV6 in terms of their efficacy to transduce primary rat astrocytes in cell culture. Such strong divergence between rAAV1 and rAAV6 tropism is unexpected, but not unprecedented. In several prior publications, these two serotypes also demonstrated quite distinct infection profiles when delivered into the brain and the heart, or used for infection of liver cells *in vitro* (Wu et al., 2006a; Markakis et al., 2010; Zincarelli et al., 2010). A likely explanation is provided by the observation on the pivotal role for a few unique amino acid residues within the AAV6 capsid components (Wu et al., 2006a). The triple mutation Y731F/Y705F/T492V in the rAAV6 capsid protein dramatically modified cell tropism and efficacy for transducing microglial cells (Rosario et al., 2016).

The significance of the present study is in identifying a new tool for manipulating gene expression in astrocytes. To date, astrocyte-specific gene transduction was reported with “canonical” rAAV5, rAAV8, and rAAV9 driving gene expression under control of the astrocytic Gfa2 promoter (see for example Young et al., 2014; Furman et al., 2016). However, astrocytic tropism of rAAV5, rAAV8, and rAAV9 may be strongly influenced by packaging or other factors. To illustrate this point, in our hands, rAAV5, rAAV8, and rAAV9 vectors acquired from ViGene Biosciences produced low-to-undetectable GFP expression in primary astrocyte cultures, but readily infected ARPE-19 cells. Other studies utilized the recently discovered preferential astrocytic tropism of rhAAV43, or the recombinant AAV species in which capsid proteins were genetically modified to increase astrocytic targeting (Koerber et al., 2009; Lawlor et al., 2009). However, even in these cases the levels of astrocytic infection were not necessarily high, with only a fraction of astroglial cells expressing transgene proteins. For example, the AAV mutants, which were engineered to enhance glial delivery, infected only ∼15% of astrocytes *in vivo* (Koerber et al., 2009). In the present work, we found very robust virus-driven GFP expression in >90% of astrocytes in the brain areas infected with rAAV6. At the very least, this is a strong starting point in design and production of more refined vectors.

Keeping in mind the high variability of the past results obtained with rAAVs from various packaging facilities, the looming question is whether the data presented in the current manuscript can be consistently reproduced by others in the field. We attempted to address these concerns through several lines of extensive *in vitro* and *in vivo* experiments. Astrocytic transduction was initially evaluated with several batches of rAAVs in the primary rat astrocytes. Viability of all “ineffective” or “less effective” rAAV constructs was validated in the same viral batches using human ARPE-19 cells, which are known to be transduced by numerous rAAVs. The cell type (astrocytic) tropism of rAAV6 was quantitatively tested *in vivo* in several animals, and the results were reproduced with different viral preparations to account for potential inter-batch variability. Finally, quantification of viral tropism was performed in a blinded fashion to minimize the chance of analytical bias. The rAAV6 constructs tested by us are available from a commercial source, where they are produced through highly standardized packaging and purification procedures. Therefore, rAAV6 can serve as a new useful tool for manipulating gene expression in rat astrocytes for all interested investigators, regardless of their prior experience with AAV constructs.

## Author Contributions

ALS, LJ, and AAM conceived study design. ALS performed experiments. ALS, DG, and AM analyzed data. YC and GG developed viral vectors; YC, GG, and LJ provided technical advice and/or help with data analysis. All authors read and corrected the final version of the manuscript.

## Conflict of Interest Statement

One of the authors, YC, has a commercial interest in ViGene Biosciences, Inc. YC did not contribute to study design or interpretation of the data. All the other authors declare that the research was conducted in the absence of any commercial or financial relationships that could be construed as a potential conflict of interest.

## Footnotes

1 http://www.vigenebio.com/

## Supplementary Material

FIGURE S1 **Comparison of astrocytic transduction by rAAV serotypes 1, 2, and 5–9 obtained from a different viral packaging batch or from a different packaging facility.** Primary rat astrocytes were infected with 10^5^ gc/cell of each serotype as indicated or not transfected (NTF). After 48 h, cells were immunostained with the anti-GFP antibody to visualize viral expression and stained with DAPI to visualize nuclei. rAAV1,2,5–9 were from ViGene Biosciences, but from a different packaging batch as compared to the data presented in **Figure 1**. Additionally, we used rAAV1 (AAV2/1), rAAV2 (AAV2/2), rAAV5 (AAV2/5), and rAAV9 (AAV2/9) constructs from the University of Massachusetts Medical School packaging facility. In these latter constructs, numerical designations stand for the inverted terminal repeat regions sequences from AAV2 and capsids proteins from AAV1, 2, 5, 9. Images were taken using an Olympus IX71 inverted microscope at 100× magnification with identical exposure settings. See “Materials and Methods” for additional information on viral composition and staining details.

FIGURE S2 **Propagation of viral transduction in the brains infected with rAAV2 or rAAV6.** Immunohistochemical images of the coronal sections from a representative animal injected with rAAV2 (**left** column) or an animal injected with rAAV6 (**Right** column). The 25-μm coronal sections were stained with anti-GFP antibody and counterstained with DAPI. Low magnification fluorescence images were captured using a Zeiss Axio Imager.M2 microscope at 50× magnification; and multiple fields from the same section were stitched together using NeuroLucida software. The anatomical hallmarks in each section are represented by drawings prepared based on the atlas by G. Paxinos and C. Watson. “The Rat Brain” (sixth edition). INSETS show matching high magnification images from the same sections, in which GFP fluorescence was captured with a Zeiss LSM510 META confocal microscope at 250× magnification (Scale bars = 50 μm). An approximate position of each inset image on coronal sections is indicated by green arrows.

FIGURE S3 **Representative images of the AAV2- **(Left)** and AAV6- **(Right)** injected rat brains captured at low magnification.** 25-μm brain sections were stained with the anti-GFP antibody to visualize viral transduction. Lower panels show digitally magnified fields from the selected areas. Red arrowheads point to representative neuronal-like cells. Yellow arrows point to cells with astrocytic morphology. Images were taken close to the injection site at identical 100× magnification and exposure settings using an Olympus IX71 inverted microscope.
